# Ultrasound and diffuse optical tomography-transformer model for assessing pathological complete response to neoadjuvant chemotherapy in breast cancer

**DOI:** 10.1117/1.JBO.29.7.076007

**Published:** 2024-07-24

**Authors:** Yun Zou, Minghao Xue, Md Iqbal Hossain, Quing Zhu

**Affiliations:** a Washington University in St. Louis, Department of Biomedical Engineering, St. Louis, Missouri, United States; b Washington University in St. Louis, Imaging Science, St. Louis, Missouri, United States; c Washington University School of Medicine, Department of Radiology, St. Louis, Missouri, United States

**Keywords:** diffuse optical tomography, pathological complete response, dual input transformer, breast cancer

## Abstract

**Significance:**

We evaluate the efficiency of integrating ultrasound (US) and diffuse optical tomography (DOT) images for predicting pathological complete response (pCR) to neoadjuvant chemotherapy (NAC) in breast cancer patients. The ultrasound-diffuse optical tomography (USDOT)-Transformer model represents a significant step toward accurate prediction of pCR, which is critical for personalized treatment planning.

**Aim:**

We aim to develop and assess the performance of the USDOT-Transformer model, which combines US and DOT images with tumor receptor biomarkers to predict the pCR of breast cancer patients under NAC.

**Approach:**

We developed the USDOT-Transformer model using a dual-input transformer to process co-registered US and DOT images along with tumor receptor biomarkers. Our dataset comprised imaging data from 60 patients at multiple time points during their chemotherapy treatment. We used fivefold cross-validation to assess the model’s performance, comparing its results against a single modality of US or DOT.

**Results:**

The USDOT-Transformer model demonstrated excellent predictive performance, with a mean area under the receiving characteristic curve of 0.96 (95%CI: 0.93 to 0.99) across the fivefold cross-validation. The integration of US and DOT images significantly enhanced the model’s ability to predict pCR, outperforming models that relied on a single imaging modality (0.87 for US and 0.82 for DOT). This performance indicates the potential of advanced deep learning techniques and multimodal imaging data for improving the accuracy (ACC) of pCR prediction.

**Conclusion:**

The USDOT-Transformer model offers a promising non-invasive approach for predicting pCR to NAC in breast cancer patients. By leveraging the structural and functional information from US and DOT images, the model offers a faster and more reliable tool for personalized treatment planning. Future work will focus on expanding the dataset and refining the model to further improve its accuracy and generalizability.

## 1 Introduction

Breast cancer, with ∼2.3  million cases annually, is the most commonly diagnosed cancer among women.^1^ Although earlier diagnosis and advances in treatment have decreased the mortality rate in most Western countries,^2^ breast cancer remains the primary cause of cancer-related death in women worldwide. Over 600,00 women died of the disease in 2023,^3^ and ∼1  million deaths are predicted for 2040.^4^ Preoperative neoadjuvant chemotherapy (NAC) is the standard-of-care for stage II and III breast cancers and is routinely used to reduce tumor size and potential metastasis, enabling breast-conserving surgery.^5^^,^^6^ Pathological complete response (pCR) is the well-validated surrogate endpoint for predicting patient NAC outcomes. However, accurately identifying patients who have achieved pCR is significantly challenging, especially in the early treatment cycles.

Breast cancer is a heterogeneous disease: ∼20% of breast cancers show amplification of the human epidermal growth factor receptor 2 (HER2+), but 10% to 20% of breast cancers lack expression of the estrogen receptor (ER), the progesterone receptor (PR), and HER2 gene amplification, a condition known as triple-negative breast cancer (TNBC). Imaging techniques to assess individual treatment responses are appealing because they are non-invasive and may provide a window of opportunity wherein ineffective treatment regimens can be altered to improve treatment outcomes. Conventional imaging methods include mammography, ultrasound (US), magnetic resonance imaging (MRI), and positron emission tomography–computed tomography (PET-CT). However, mammography has low sensitivity in evaluating the response to NAC.^7^ US is moderately accurate and has the additional benefits of easy access and low cost.^8^^–^^11^ MRI and PET-CT have both demonstrated good accuracy (ACC) in predicting pCR.^12^^–^^14^ However, repeated MRI and PET-CT imaging during NAC are very expensive.

Diffuse optical tomography (DOT) and spectroscopy using near-infrared (NIR) diffused light have been explored to predict and monitor tumor vasculature response to NAC.^15^^–^^27^ The NIR technique utilizes intrinsic hemoglobin contrast, which is directly related to tumor angiogenesis. It is particularly effective in mapping earlier tumor angiogenesis changes during NAC. However, DOT using pure NIR light suffers from intense light scattering that hinders lesion localization. To overcome the location uncertainty, our group developed US-guided DOT,^28^ a unique approach that employs a commercial US transducer and NIR optical imaging sensors mounted on a hand-held probe. The lesion structure information provided by the co-registered US aids the optical imaging reconstruction and thus reduces the location uncertainty and improves the quantification accuracy of the light. Furthermore, DOT can be easily integrated with US systems for dual-modality assessment of breast cancer response to NAC.^18^^,^^19^^,^^22^^,^^23^

Recent developments in artificial intelligence and radiomics have enhanced the effective prediction of tumor treatment, with US offering a cost-effective, practical, and radiation-free option, even though US is moderately accurate. Approaches such as deep learning radiomics models use US images at multiple NAC treatment points for better prediction. Yet, these methods are limited by their reliance on post-analysis and lack of end-to-end modeling, which restricts their learning capabilities and flexibility.^29^^–^^33^

The introduction of transformers in natural language processing, a deep learning model based on a multi-head self-attention mechanism, has now extended to computer vision, including image classification and enhancement. Vision transformers (ViTs) represent each image as a token sequence, utilizing the global dependence between image tokens for more effective analysis. This advancement marks a significant stride in applying sophisticated artificial intelligence techniques for more precise and effective breast cancer diagnosis and treatment evaluation.^34^^,^^35^

Predicting pCR using deep learning methods has been extensively studied. For example, Joo et al.^36^ utilized a multimodal deep learning approach, combining clinical information with pretreatment MR images, to highlight the method’s efficacy in enhancing prognostic accuracy through integrated analysis of diverse data types. Tong et al.^37^ developed a dual-input transformer (DiT) model, optimized with four specialized modules for analyzing US images, to predict NAC effectiveness in breast cancer. Wu et al.^38^ deployed a UNet model to handle data before treatment, cycle 1, and before surgery to extract features and predict pCR. However, these models utilize only single modality images to predict pCR and have achieved moderate ACC. In this study, we design a deep-learning DiT model that uses co-registered US and DOT images. The structural information in US images and the functional information in DOT images are more accurate in predicting pCR than the information from a single modality alone. To achieve this, we modified the DiT model, originally designed only for US images, to use US images, DOT reconstruction images, and tumor receptor biomarkers. To the best of our knowledge, this system embodies the first attempt to predict pCR using US and DOT images with an advanced deep-learning model.

## 2 System

The co-registered US and DOT system features a hand-held probe equipped with four laser diodes with wavelengths of 730, 785, 808, and 830 nm. These diodes are modulated at 140.02 MHz and operated sequentially across nine source positions on the probe. This setup utilizes a heterodyne detection method where the detected signals, after interaction with the tissue, are mixed with a 140 MHz reference signal, resulting in a demodulated 20 kHz signal. At its core, the probe incorporates a US transducer to provide co-registered B-scan US images, while 14 photomultiplier tube detectors, connected via light guides, simultaneously capture the diffuse reflectance. The DOT system was designed for rapid data collection in 3 to 4 s for each complete data acquisition from all sources, at source-detector distances ranging from 3.2 to 8.5 cm. Multiple data sets were acquired at the tumor location and contralateral symmetric location, which were used as a reference.

## 3 Dataset

A total of 60 patients with NAC were included in this study, each undergoing US imaging and DOT reconstructions across four time points.^18^^,^^19^ The studies were approved by local Institution Review Boards and were Health Insurance Portability and Accountability Act compliant. All patients signed the informed consent. Initially, patients underwent baseline pre-NAC scanning, followed by subsequent scans at 2- to 3-week intervals depending on treatment regimens, constituting cycles 1 to 3. Pre-NAC and cycles 1 to 3 data were collected to facilitate early prediction of treatment response. Table 1 lists details of the cancer biomarker types, age, and final pathology based on surgical specimens from a total of 60 patients. Miller-Payne (PM) grades were used for the assessment of response: PM 4 to 5 were grouped as responders, and PM 1 to 3 were non-responders.^18^^,^^19^

**Table 1:** Patient information.

Receptor type	Age			Miller-Payne grade (no. of patients)		Tumor type (no. of patients)	
	Mean	Min	Max	4 to 5	1 to 3	IDC	ILC
HER2+, ER+/−	48.2	30	74	21	4	23	2
HER2−, ER+	49.2	31	72	3	13	14	2
HER2−, ER-	51.4	24	71	11	8	18	1

HER2+, HER2 positive tumor; ER+, estrogen receptor positive tumor; IDC, invasive ductal carcinoma; ILC, invasive lobular carcinoma

**Fig. 1:**
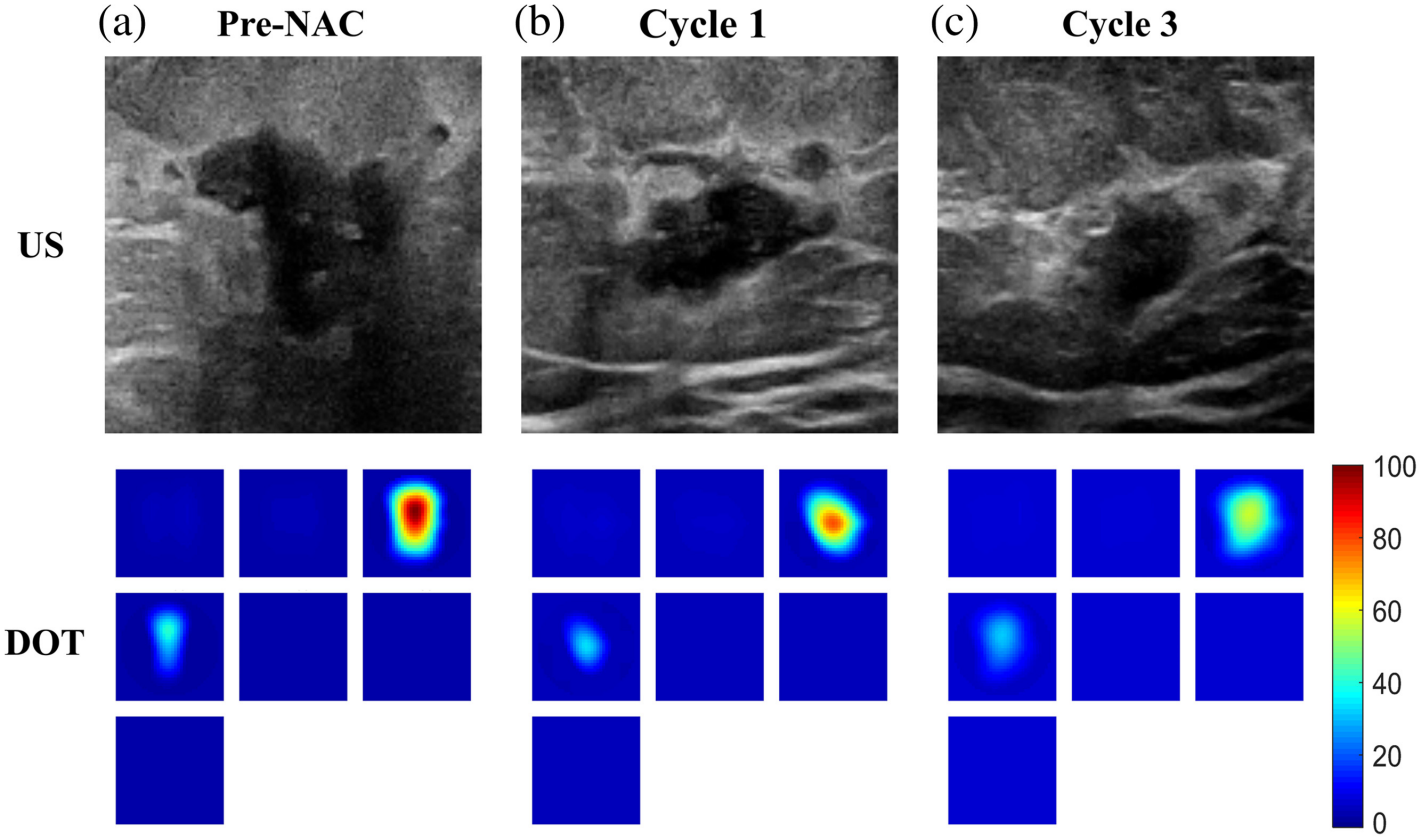
Twenty-four-year-old patient with a 3.4 cm diameter triple receptor-negative cancer. The top row shows US images; the lower row shows DOT images. (a) Images pre-NAC, (b) images after cycle 1 treatment, and (c) images after cycle 3 treatment. Each DOT image has seven slices from left to right and top to bottom representing x−y spatial image of 9  cm×9  cm with 0.5 cm in depth from the skin surface to 3.5 cm depth.

Figure 1 presents the case of a 24-year-old patient who had TNBC cancer treated with six cycles of NAC. US imaging reveals a significant reduction in tumor size from the first to third treatment cycles. In addition, DOT imaging indicates a decrease in total hemoglobin levels. This overall reduction in both tumor size and hemoglobin level characterizes a positive response to NAC. The final surgical pathology revealed that the patient received a pCR with no residual tumor left (PM 5).

**Fig. 2:**
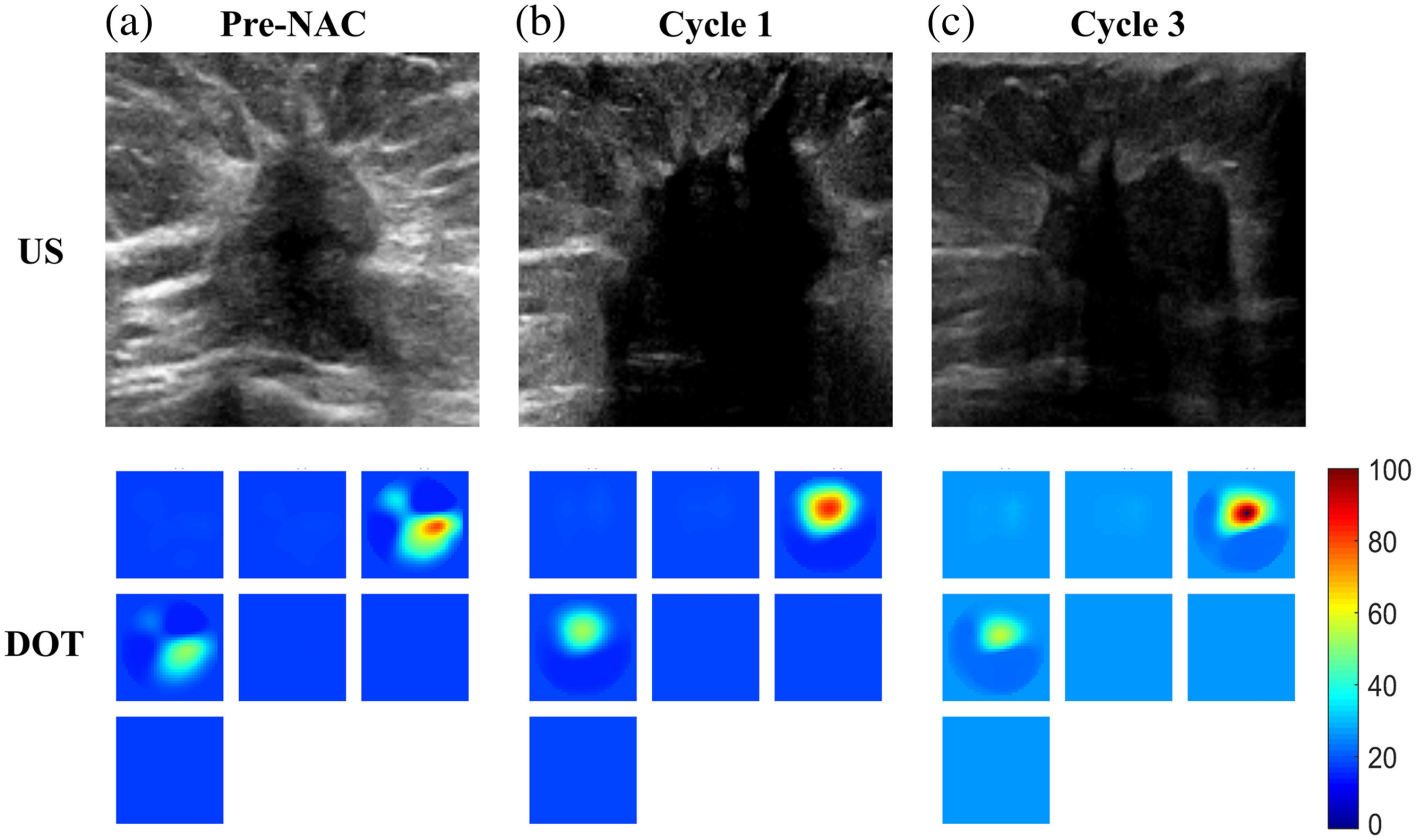
Fifty-two-year-old patient with a 6.8 cm diameter tumor. Panels (a)–(c) and rows have the same information as in Fig. 1.

Conversely, Fig. 2 depicts a non-responder case of a 52-year-old woman who had ER+, PR−, and HER2+ cancer treated with six cycles of NAC. Here, US imaging shows highly irregular shapes and an increase in tumor size due to treatment scar from cycle 1 to 3 images. The corresponding DOT images reveal no reduction in total hemoglobin level; in fact, the level has increased slightly from cycles 1 to 3. This lack of therapeutic response, evidenced by both imaging modalities, categorizes the patient as a non-responder. The surgical pathology report revealed that the patient did not respond to NAC, with a residual tumor measuring 1.6 cm.

**Fig. 3:**
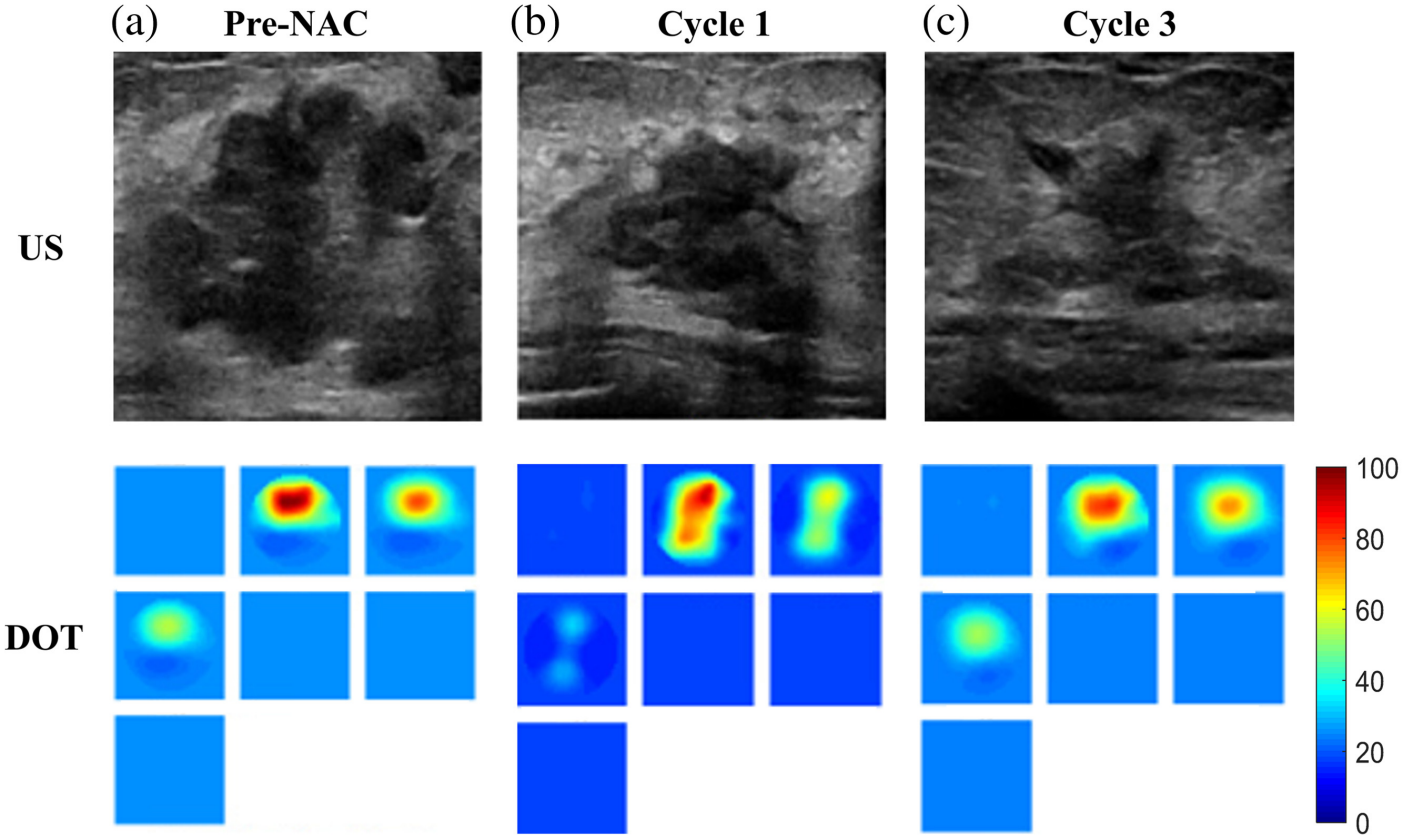
Fifty-six-year-old patient with a 4.1 cm diameter cancer measured by US. Panels (a)–(c) and rows have the same information as in Fig. 1.

Figure 3 is an example that presents a more challenging scenario for assessment. When examining the images from the US, it appears that the lesion is diminishing in size. However, the DOT images reveal that the lesion hemoglobin level is high. Subsequent pathology results indicate that the patient did not respond to treatment, with a residual cancer measuring 2.4 cm as revealed by surgical pathology.

## 4 Methodology

### 4.1 DiT Model

The DiT model consists of three sections: (a) isolated token-to-token (T2T) patch embedding, (b) shared position and time embedding modules, and (c) weighted average pooling feature representation (WAPFR). Here, we used six images as a group, which included US pre-NAC, cycle 1, and cycle 3 images, and DOT pre-NAC, cycle 1, and cycle 3 images. Then, we went through three sections of the DiT model to obtain the final prediction of the responder or non-responder. Details of each section are given as follows:

The input US images are sized at 128×128  pixels. For the DOT images, we reconstruct the 3D volume and visualize it using seven slices, with each slice a size of 37×37  pixels, which corresponds to 9 cm by 9 cm in spatial dimensions and 0.5 cm in depth. To transform these slices into the model’s input format, we rearrange them into a 111×111 matrix by placing the slices side by side. Finally, we resize this matrix to 128×128  pixels to match the resolution of the US images.

The transformer architecture in our model is configured with specific parameters to optimize its performance. The input and output dimensions are set to 64. The depth of the model is 8, meaning eight transformer layers. Each layer employs 16 heads for multi-head self-attention. The dimension per head is 64. In addition, the model includes a multi-layer perceptron with a dimension of 64 for the feedforward network within each transformer block, which processes the attention outputs.

#### 4.1.1 Isolated T2T patch embedding

**Fig. 4:**
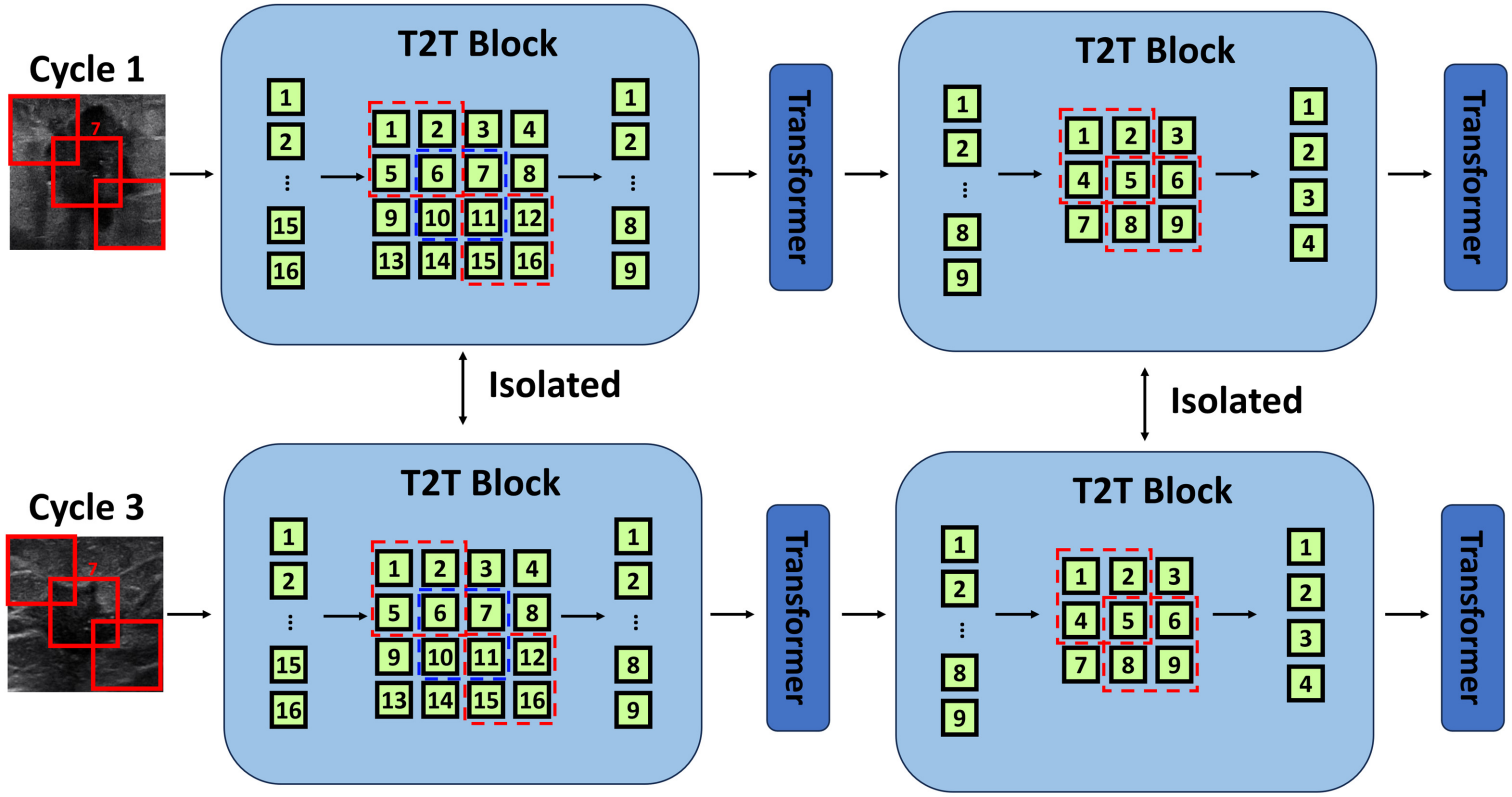
Image patching and T2T block.

This method first uses progressive tokenization based on a T2T module, as shown in Fig. 4. A total of 16 overlapping patches are generated, enabling the model to learn from complex relationships amongst different regions. The T2T module’s progressive tokenization allows for multi-level feature extraction, facilitating the capture of both local and global features. This method reshapes the 16 patches to form an image and regenerates concatenated nine patches using a 2×2 kernel to feed into the transformer layer. Using the same approach, the nine patches are reshaped using a 2×2 kernel to regenerate four patches to feed into the next transformer layer. This hierarchical tokenization process facilitates the extraction of both fine-grained details and broader contextual information, leading to a more detailed representation of the image.

The T2T block can extract more levels of features than the conventional patching method due to its ability to progressively tokenize images and retain more structural information. Unlike conventional patching that divides an image into non-overlapping patches in a single step, the T2T module’s progressive tokenization allows for multiple levels of patch generation and transformation. This results in a richer and more diverse feature set, combining finer details with broader patterns and leading to a more robust representation of the input images.

At different time points, the structure or texture may differ for different cycles, so we use triple-isolated T2T modules for learning fusion operations at specific time points, favoring soft split tokenization to retain more structural information. The isolated T2T modules ensure that structural information, particularly around regions of interest such as tumors, is preserved. This method also considers the region of interest around the tumor, accommodating varying scanning views while maintaining relative positions. This adaptability helps in retaining consistency and reliability in feature extraction across different time points, enhancing the model’s ability to detect and analyze intricate details within the images.

#### 4.1.2 Shared position and time embedding modules

These modules enhance the model’s capability to interpret spatial and temporal data from US and DOT images. The shared position embedding uses a learnable matrix to encode spatial relationships of pre-NAC and cycle 1 and 3 image tokens. The time embedding module distinguishes tokens from different time points, aiding in effective temporal information utilization, crucial for tracking treatment responses.

#### 4.1.3 WAPFR

**Fig. 5:**
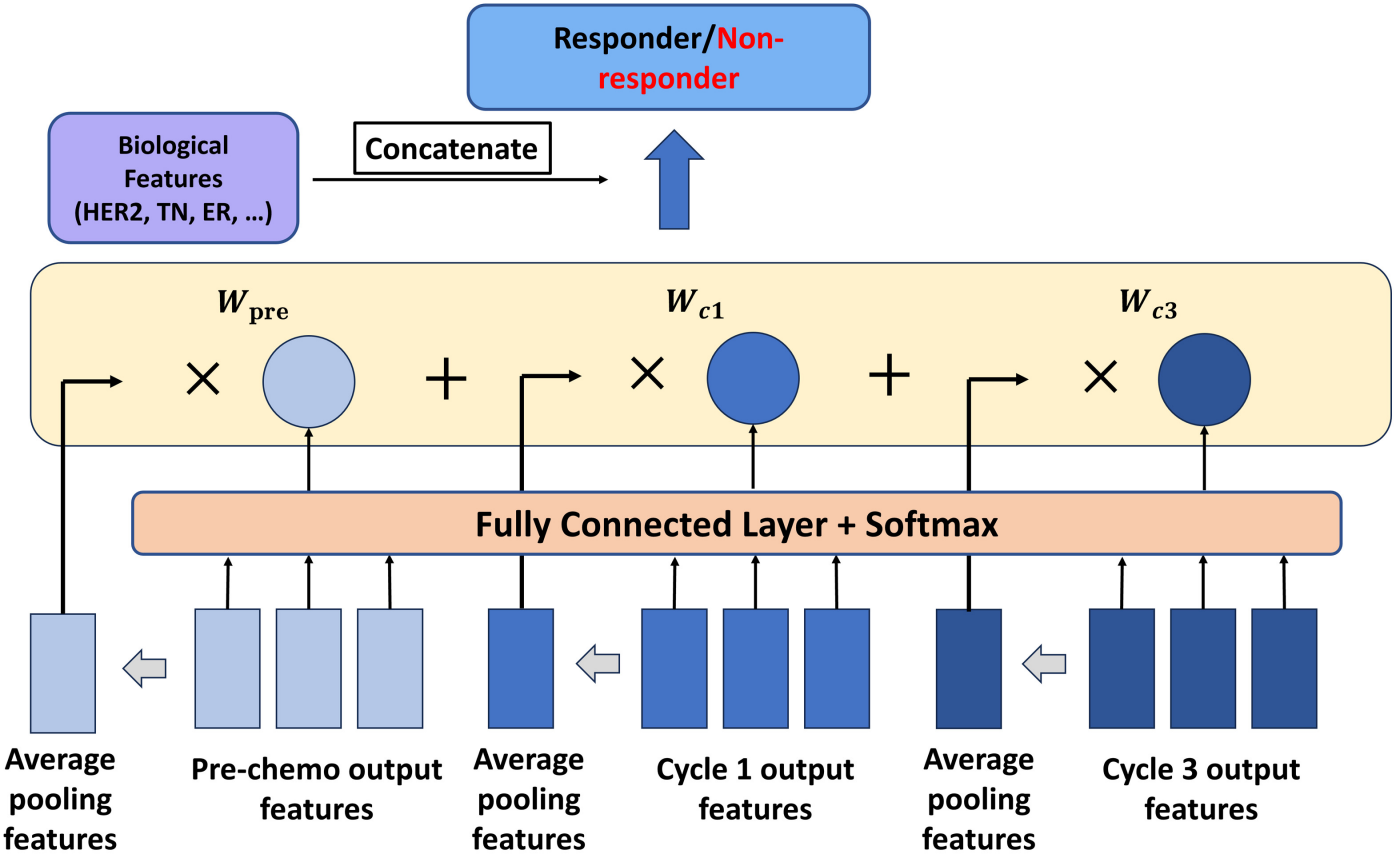
WAPFR structure. The first layer averages pooling for each image’s output features to get the average features. The output features also go through the fully connected layer and softmax layer to generate weights for each input image. Then, multiply with each average feature and concatenate with tumor marker features to predict the final output.

This component starts with average pooling on output tokens along the sequence and embedding dimensions, creating image and patch feature representations, respectively. It then uses fully connected and softmax layers for weight determination, crucial for weighting image features from different time points, thereby enhancing the classification process, as shown in Fig. 5.

In this study, we collect several tumor features, including invasive lobular carcinoma (ILC), invasive ductal carcinoma (IDC), and tumor grade, to assess the tumor characteristics. The study also considered breast cancer subtypes, such as TNBC, HER2 status, and ER status, to provide a comprehensive evaluation of the tumor biology. Then, we concatenate these features with the weighted imaging features and place them to a final, fully-connected layer to predict the response.

### 4.2 Ultrasound-Diffuse Optical Tomography (USDOT)-Transformer

**Fig. 6:**
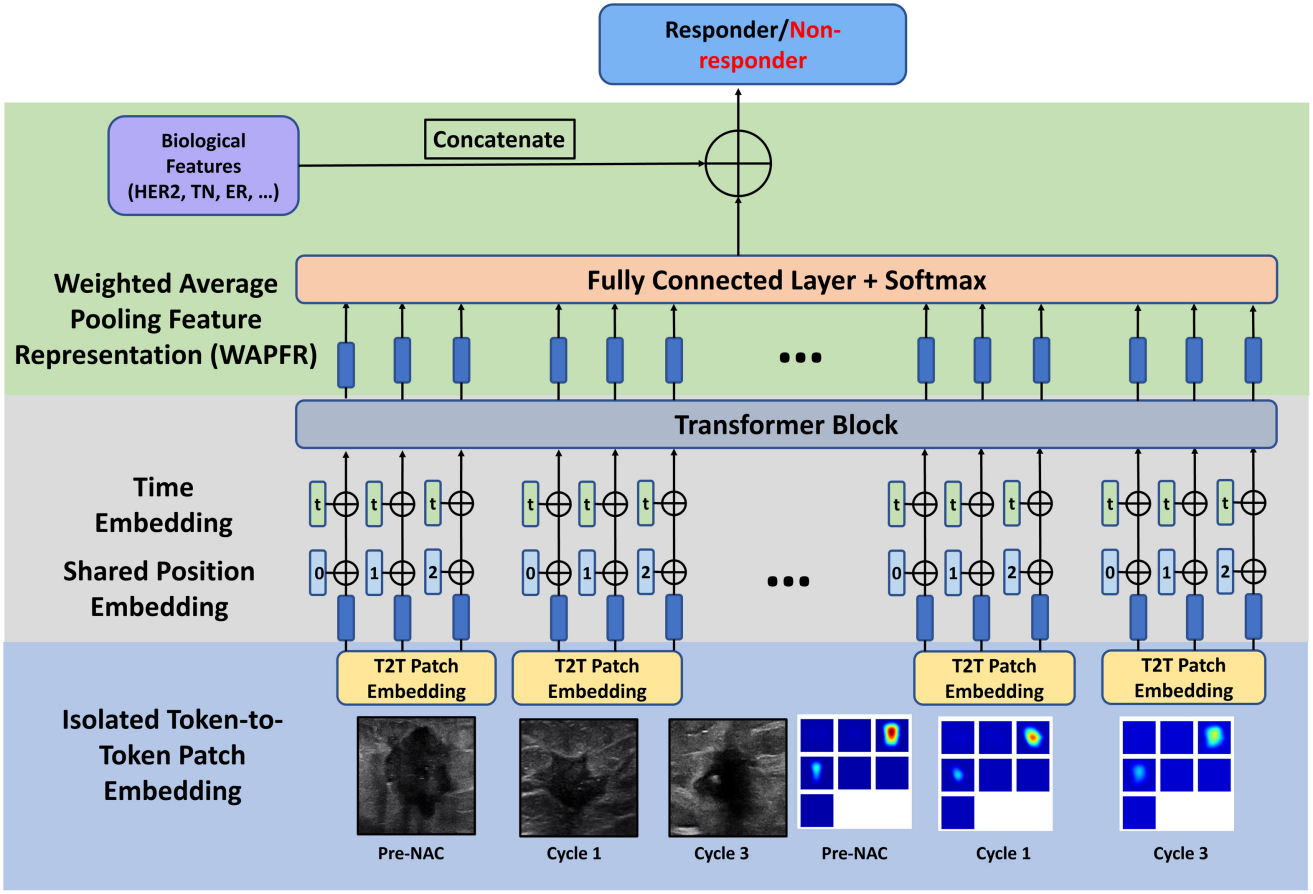
USDOT-Transformer structure.

In our study, we enhanced the DiT model to incorporate both US and DOT images, as depicted in Fig. 6. This modification involved not only extracting features from US images but also integrating a transformer block for DOT feature extraction. Prior to the final softmax layer, tumor marker features were concatenated to enrich the model’s analysis.

We chose the modified transformer model for this project due to its groundbreaking impact on natural language processing and its recent success in computer vision tasks. The transformer’s self-attention mechanisms capture long-range dependencies and global context more effectively than traditional convolutional neural networks (CNNs), which is particularly beneficial for medical imaging where spatial relationships within the image are crucial for accurate diagnosis. In addition, the transformer can integrate multimodal data such as US and DOT images. This capability aligns perfectly with our goal of integrating US structural and DOT functional imaging data. Given the complexity and importance of accurately predicting a pCR in breast cancer patients, the transformer’s advanced self-attention mechanisms and sequential data processing provide a robust framework for capturing intricate patterns in medical images, leading to a more comprehensive assessment of tumor response to NAC and improved personalized treatment planning.

In addition, because the DOT images are low-resolution function images when we extract the features from them, it is easy to get overfit. The most relevant feature to predict pCR is the maximum value within the tumor area. Therefore, in addition to using DOT images as input, we also calculated the maximum value for each DOT image as additional input. Thus, the final output is predicted from US features, DOT images and features, and tumor markers.

However, this adaptation presented a challenge: the increase in input image combinations. Originally, the DiT model handled combinations from two image types (US pre-NAC, cycle 1 and 3). With our modification, this expanded to six types (US/DOT pre-NAC, cycle 1, cycle 3), resulting in an exponential increase in data combinations. For instance, considering 10 images for each modality and cycle across 10 patients, the original DiT model would process 1000 combinations (10×10×10). In contrast, our USDOT-Transformer model faced a staggering one million combinations. This significant increase necessitated much longer training time for the model.

**Fig. 7:**
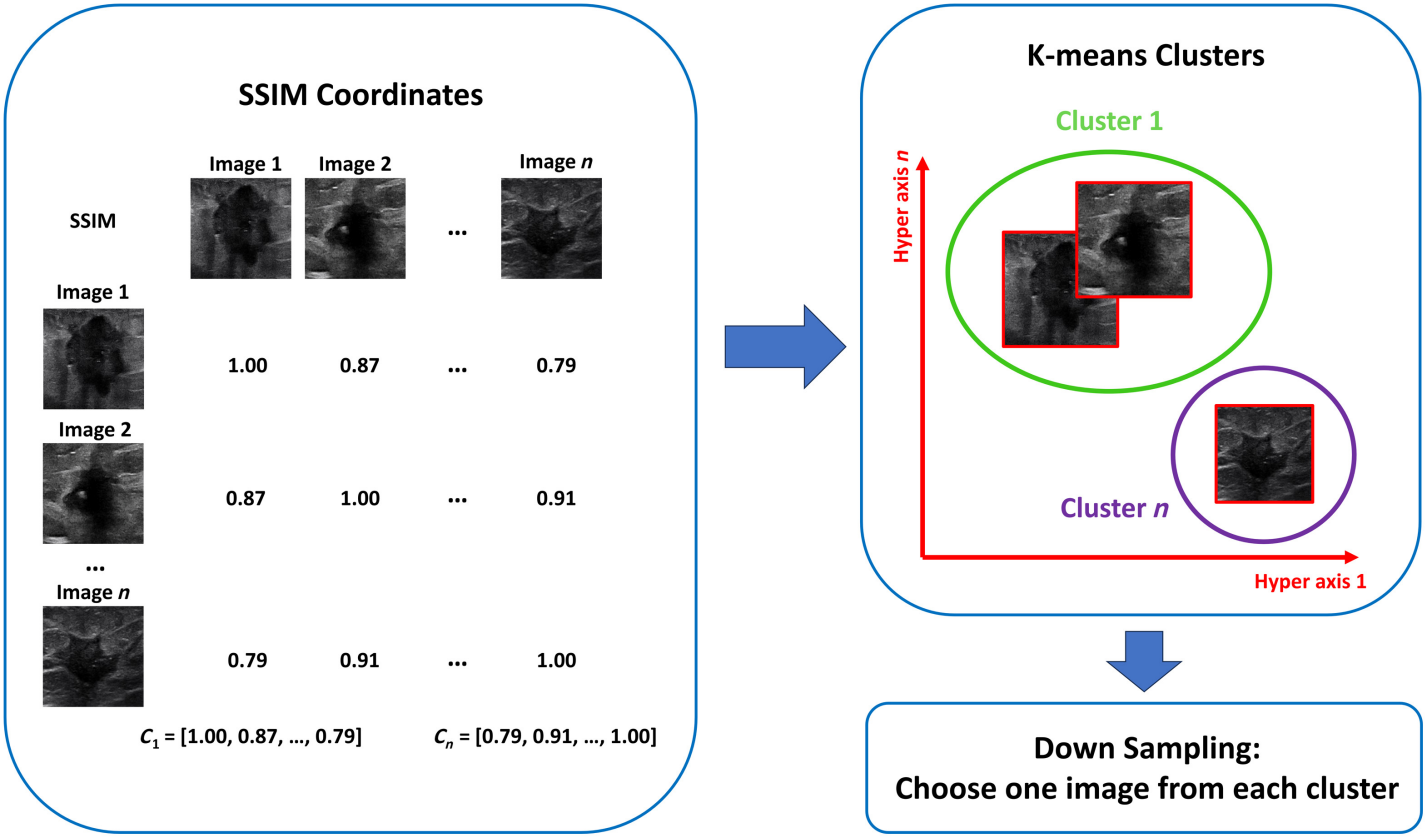
Image down-sampling procedure.

To address this challenge, we implemented a downsampling method, leveraging the fact that at each time point, similar measurements are obtained multiple times. Our first step in reducing redundancy was to calculate the similarity between images in each cycle using the structural similarity index (SSIM), as illustrated in Fig. 7.

Upon determining the SSIM values for each image, we then applied the K-means algorithm to identify five central points from all images. By focusing on these central images and removing the others, we efficiently downscaled the dataset without significant information loss.

## 5 Results

### 5.1 Model Development

The USDOT-Transformer model was trained on an RTX 2080Ti GPU using 100 epochs. To optimize the training process, we employed the Adam optimizer and implemented the ReduceLROnPlateau scheduler to avoid overfitting. The loss function used was binary cross-entropy with a learning rate set at $4\times 10^{-5}$. We set the batch size to 24 and applied a weight decay of $10^{-6}$. Utilizing a cross-validation strategy, we fine-tuned the hyperparameters, and we used the entire dataset to train the final model. The total number of trainable parameters in the USDOT-Transformer model is ∼42  million.

### 5.2 Statistics Analytics

Our study utilized a fivefold cross-validation approach to evaluate the performance of the USDOT-Transformer model, and the results underscore its potential in predicting pCR to NAC in breast cancer patients. The average area under the receiving characteristic curve (ROC) (AUC) across five models was remarkably high, with AUC=0.96 [95% confidence interval (CI): 0.93 to 0.99], suggesting that the excellent model accuracy in distinguishing between patients is likely to achieve pCR from those who are not.

The performance of each model variant is detailed in Table 2, where the AUC values range from 0.9137 to 1.0000 across five different folds. This variance highlights the model’s robustness and consistency in processing complex patient data to predict treatment outcomes. Such predictive capability is critical for personalizing breast cancer treatment, enabling clinicians to optimize therapeutic strategies based on predicted responses.

**Table 2:** AUC results for the USDOT-Transformer model.

Model 1	Model 2	Model 3	Model 4	Model 5
1.0000	0.9306	0.9214	0.9409	0.9514
0.9935	0.9741	0.9723	0.9881	0.9211
0.9615	1.0000	0.9541	0.9805	1.0000
0.9455	0.9255	1.0000	0.9924	0.9266
0.9137	0.9433	0.8974	0.9375	0.9521

**Fig. 8:**
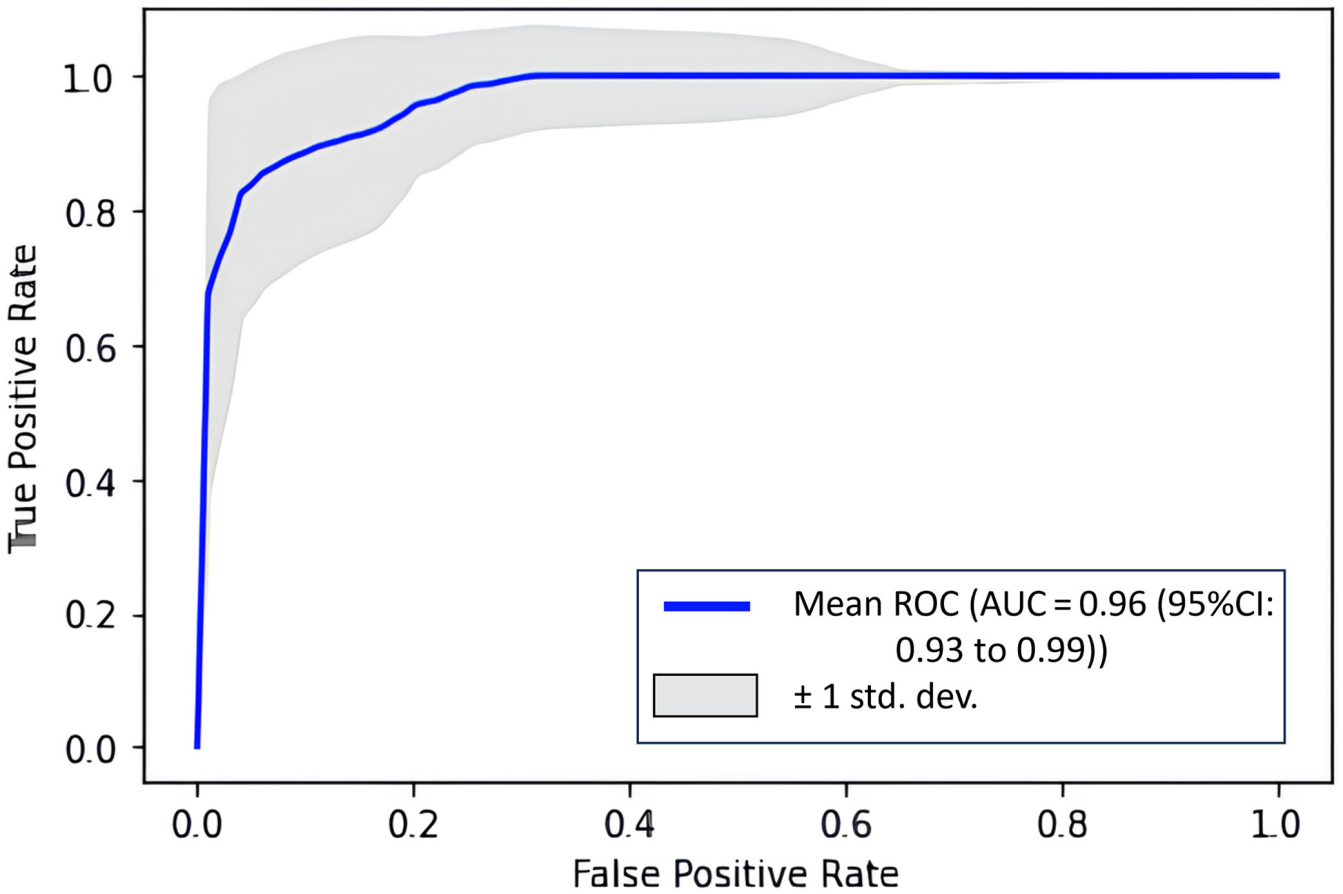
ROC for USDOT-Transformer model.

We also draw the average ROC for the USDOT-Transformer model with five times fivefold cross-validation in Fig. 8. The model performed well as compared with the US- or DOT-only model in ablation studies.

## 6 Ablation Studies

### 6.1 Model Selection

To determine the best model for this project, we have chosen several baseline models for comparison. Here, the first model is ResNet-50,^39^ which is widely used and has a similar number of trainable parameters compared with ViT and our model. We use different training datasets as input, pre-NAC, cycle1, and with or without cycle 3 data. In addition, we include the tumor features for a fair comparison. The features are concatenated in the fully connected layer. The second model we chose is ViT, which is the basic model within our USDOT-Transformer model. We also use different datasets and train without the tumor features.

Table 3 shows the AUC and ACC results for different comparison studies. ResNet-50 performs well but shows lower accuracy and AUC compared with transformer models, particularly when tumor features are included. The ViT outperforms ResNet-50, especially with data from multiple treatment cycles (pre-NAC, cycle 1, cycle 3), and further improves with the inclusion of tumor features, demonstrating its capability to integrate diverse data types. Achieving the highest AUC and ACC across all configurations, the USDOT-Transformer’s superior performance in integrating US and DOT images with tumor biological features significantly enhances predictive accuracy, which justifies its selection. The USDOT-Transformer predicts a pCR in breast cancer patients undergoing NAC.

**Table 3:** Results of comparison models with different datasets and with or without biological features.

Method	Dataset	Tumor features	AUC (95% CI)	ACC (95% CI)
ResNet-50	PC, C1^a^	No	0.844 (0.897 to 0.884)	0.817 (0.781 to 0.849)
ResNet-50	PC, C1, C3^a^	No	0.871 (0.848 to 0.893)	0.854 (0.832 to 0.883)
ResNet-50	PC, C1, C3	Yes	0.883 (0.852 to 0.906)	0.856 (0.818 to 0.894)
ViT	PC, C1	No	0.864 (0.800 to 0.915)	0.858 (0.833 to 0.895)
ViT	PC, C1, C3	No	0.914 (0.853 to 0.959)	0.907 (0.872 to 0.947)
ViT	PC, C1, C3	Yes	0.937 (0.886 to 0.975)	0.922 (0.885 to 0.974)
USDOT-Transformer	PC, C1, C3	No	0.931 (0.909 to 0.962)	0.933 (0.911 to 0.957)
USDOT-Transformer	PC, C1, C3	Yes	0.958 (0.927 to 0.990)	0.947 (0.932 to 0.984)

a PC, C1, and C3 represent pre-NAC, cycle 1, and cycle 3, respectively.

### 6.2 Input Dataset Selection

To validate that our model is optimal, we have done several ablation studies. First, we built three models, US-only, DOT-only, and USDOT-Transformer models. In addition, we tested the effect of including data from different treatment cycles for pCR prediction. To control the variable, for each model, we only use pre-NAC data and one of the three cycles. The results in Table 4 show that the US-only and DOT-only models cannot achieve high accuracy for pCR compared with the USDOT-Transformer model. DOT predicts well using pre-NAC and cycle 3 data, whereas US predicts well using pre-NAC and cycle 1 data.

**Table 4:** AUC results for dual input model using US-only, DOT-only, or US and DOT data.

AUC	Pre-NAC and cycle 1	Pre-NAC and cycle 2	Pre-NAC and cycle 3
US only	**0.8802**	0.8050	0.8592
DOT only	0.8012	0.7930	**0.8237**
US and DOT	**0.9428**	0.8275	0.9172

Bold values indicate the highest AUC achieved for each dataset configuration.

**Fig. 9:**
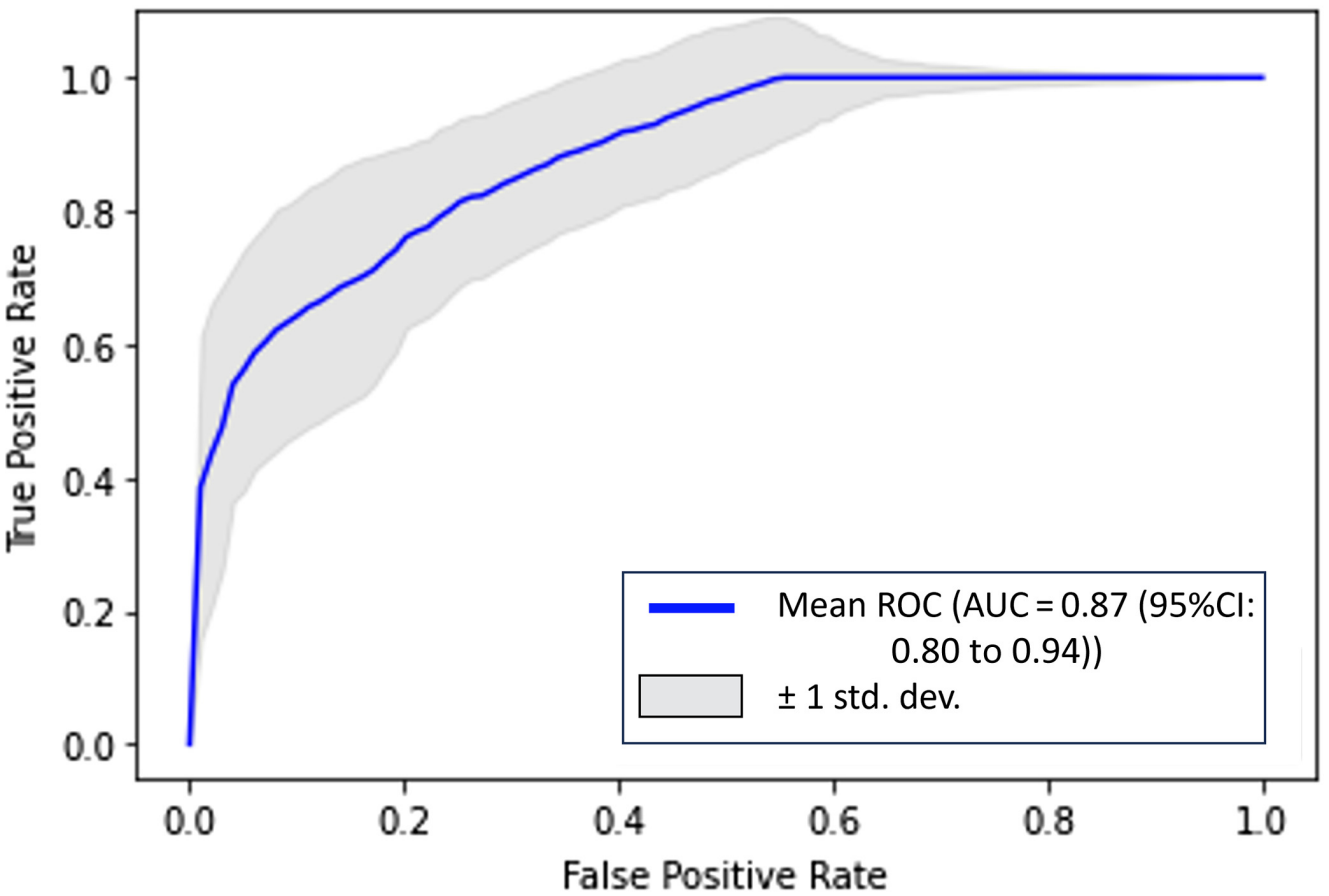
Average ROC for US-only model.

**Fig. 10:**
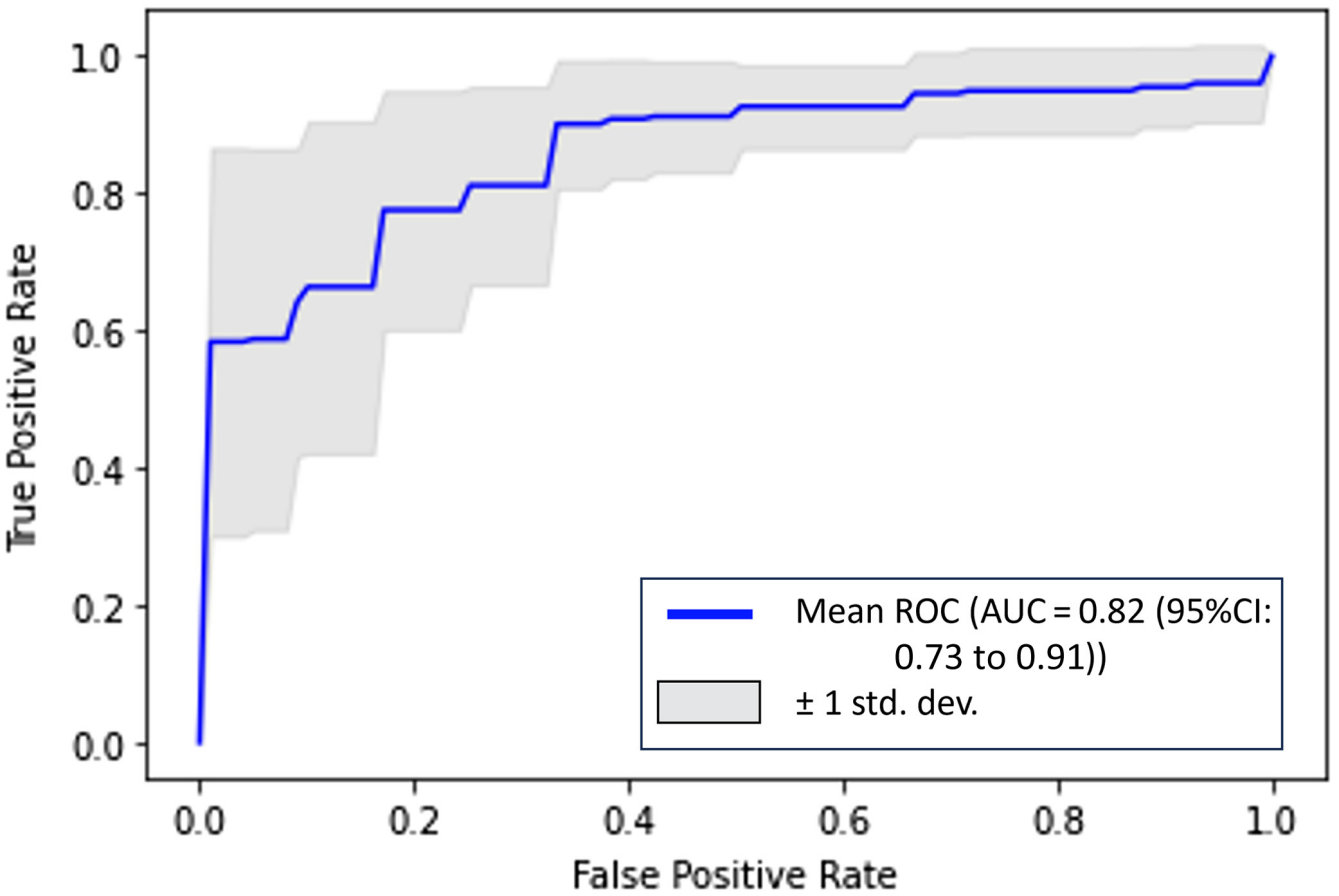
Average ROC for DOT-only model.

In Figs. 9 and 10, we plotted the fivefold average ROCs for the US-only model and DOT-only model. Compared with the USDOT-Transformer model, the AUC values for US only and DOT only are much lower, which suggests that the USDOT-Transformer model learned both features from DOT and US images.

### 6.3 Patch Size Selection

The selection of patch size in our model significantly affects its performance. We used four different size patches to validate the model’s performance, adjusting the batch size to 12 for fair comparison and to prevent out-of-memory errors with larger models.

Generally, smaller patches capture finer details and local features within the image, which is crucial for tasks requiring high spatial resolution and precise localization. Larger patches, on the other hand, make it easier for the model to integrate global context and are less sensitive to noise.

Table 5 shows the prediction performance and training time for different patch sizes. As observed, smaller patch sizes yield better AUC and ACC, with the highest being 0.967 and 0.957, respectively, for the 3×3 patch size. However, this comes at the cost of significantly longer training times. Balancing performance, model complexity, and training time, we chose a 7×7 patch size for our final model.

**Table 5:** Model’s performance with different patch sizes.

Patch size	AUC	ACC	Training time (h)
3	0.967	0.957	55
5	0.964	0.955	37
7	0.958	0.948	25
9	0.931	0.934	20

## 7 Discussion and Future Directions

Currently, the standard of care (SOC) NAC is based on tumor receptor HER2, ER, and TNBC status to determine the treatment regimens and number of cycles used. Many clinical trials may use advanced imaging, such as PET, PET-CT, and MRI, to assess response. However, due to the expensive cost, these modalities are not used in SOC.^40^^,^^41^ Our earlier publication showed that the receptor-only biomarkers of these 60 patients only provided an AUC of 0.799 (95% CI: 0.688 to 0.910), which was much lower than that of the USDOT-Transformer reported in this study.^19^

The USDOT-Transformer model exhibits competitive performance when compared with conventional logistic regression models,^18^^,^^19^ notably in its ability to automatically extract and analyze features from US and DOT images. Traditional models often require expert inputs from US and DOT measurements, a process that is prone to variability among operators. The automation provided by the USDOT-Transformer model represents a significant advancement in streamlining the prediction of pCR, potentially speeding up clinical decision-making and reducing the burden on healthcare professionals.

Our findings demonstrate the superiority of combining US and DOT imaging modalities over using either modality alone. This multimodal approach leverages the structural information available in US images and the functional insights from DOT images, leading to a more comprehensive assessment of tumor response to chemotherapy. This integrative analysis significantly improves the prediction accuracy of pCR, underscoring the value of combining diverse data types in medical imaging.

In our study, we compared the performance of the proposed DiT model with a traditional CNN-based model, specifically ResNet-50. Our findings indicate that transformer-based models outperformed the CNN model, particularly due to their advanced self-attention mechanisms. These mechanisms provide a unique form of interpretability by highlighting the global interconnections within the data, rather than focusing solely on local features as CNNs typically do. This global perspective is crucial for medical imaging tasks where understanding the broader context of tissue structures and their interactions across an image can lead to more accurate diagnoses. DiT and ViT excel in capturing these wide-ranging patterns, potentially offering new insights into complex medical conditions that manifest across extensive areas of an image. For instance, in predicting pCR in cancer treatment, understanding the entire tumor environment and its interaction with surrounding tissues can be critical.^42^

Next, the generalizability of the USDOT-Transformer model is supported by its ability to handle variations in imaging data and patient characteristics. Despite being trained on a relatively small dataset of 60 patients, the model achieved a mean AUC of 0.96 from a fivefold cross-validation. This suggests that the model has learned robust features that generalize well to different subsets of the data. However, expanding the dataset to include more patients is essential to test the robustness of the model.

Several areas need further exploration to enhance the USDOT-Transformer model. First, due to concerns over training time and memory constraints, the analysis excluded cycle 2 data. Including these data can provide a more nuanced understanding of treatment response over time, although it requires increased computational resources. Future work should explore efficient ways to incorporate this additional time point. Second, to reduce the model size and the need for input datasets, we can design two separate models. One is the US model using only pre-NAC and cycle 1 data, and the other is a DOT model using only pre-NAC and cycle 3 data. We can generate the final output by the weighted sum of each model’s output. Finally, developing methods for automating real-time image inputs and prediction can assist oncologists in making timely decisions for personalized treatment planning.

In summary, the USDOT-Transformer model represents a significant step forward in the dual-modality US- and DOT-based prediction of pCR of breast cancer patients to NAC. Its ability to integrate multimodal imaging data through advanced deep-learning techniques offers a promising avenue for personalizing cancer treatment. Future studies should focus on addressing the identified limitations. By advancing these areas, we can move one step closer to personalized treatment and improving outcomes for breast cancer patients.

## Data Availability

The code and data are available on GitHub: https://github.com/OpticalUltrasoundImaging/breast_dit. Data underlying the results presented in this paper are not publicly available at this time but may be obtained from the authors upon reasonable request.
